# Dutch postgraduate GP selection procedure; reliability of interview assessments

**DOI:** 10.1186/1471-2296-14-43

**Published:** 2013-03-27

**Authors:** Margit I Vermeulen, Marijke M Kuyvenhoven, Nicolaas P A Zuithoff, Yolanda van der Graaf, Roger A M J Damoiseaux

**Affiliations:** 1 Julius Centre for Health Sciences and Primary Care, University Medical Centre Utrecht, Utrecht, The Netherlands

## Abstract

**Background:**

Semi- structured interviews are the core of the Dutch selection procedure for postgraduate general practice (GP) training. A staff member, trainer and trainee independently assess personal qualities. Aiming to improve the selection procedure we were interested in the reliability aspects of these interviews. We investigated the inter-rater reliability of the interview for groups of two or three assessors and the degree to which candidates’ characteristics and qualities assessed during interviews explained admission into GP training, controlled for differences between those who apply for the first versus the second or third application.

**Methods:**

An observational study was conducted of all candidates who entered the Utrecht selection procedure between April 2008 and 2010. Candidates’ characteristics and qualities were collected. Inter-rater reliability of different compositions of the interview group per quality was estimated. Factors associated with admission into GP training were assessed.

**Results:**

The study population included 394 candidates. Twenty-six candidates were rejected based on their application letter (4.4%). Three candidates who applied more than 3 times were excluded. Ultimately, 206 of the 365 candidates were admitted to the GP training (56,4%). The inter-rater reliability was satisfactory (ICC: 0.78 – 0 .84). Reduction from three to two assessors slightly reduces the ICC. The candidates’ qualities independently explained admission to GP training, whereas individual characteristics did not. These results did not differ for candidates who applied for the first time versus candidates applying for the second or third time.

**Conclusion:**

Selection interviews with two assessors yielded a satisfactory level of reliability. Individual characteristics were not associated with admission, whereas scores related to candidate qualities did show such an association. The results of those applying for the second or third time were similar.

## Background

The core of the present Dutch selection procedure for postgraduate general practice (GP) training includes semi-structured interviews. These personal interviews are conducted by a staff member, a trainer and a trainee to assess candidates’ motivation, orientation on the job, learning needs and personal attributes. Comparable selection methods are used in many European countries, originating from the discipline based training model developed in the last quarter of the 20th century [1]. In general, the reliability of interview assessments in medical school admission is considered moderate to good. Reliability increases by structuring interviews, training assessors and increasing the number of assessors or interviews [2-5].

However, this assessment method can be criticised from different points of view. First, it weakly predicts future clinical and academic performance [3,4,6,7]. In addition, we have recently found that the current interview procedure yields doubts about fairness for candidates, and the respective departments of choice have a strong influence on admission [1].

Given these considerations, the national Dutch GP training (Huisarts Opleiding Nederland) aims to update the selection to a competency- based procedure with an extension of instruments [8]. As we decided to maintain a highly structured interview in the new procedure, we investigated the reliability of interview assessments in the current procedure with three groups of assessors (staff members, trainers and trainees). From an economic perspective, we explored the degree to which reliability diminishes in case of reduction from three to two interview assessors. Another aim of this study was to determine whether our earlier findings, that individual characteristics such as age and gender, do not predict admission into GP training, could be replicated. In addition, we explored whether the results differed for candidates who applied for the first time versus the second or third time [1]. The data for this study are the routinely registered data of the selection procedure on the department of Utrecht from 2008–2010.

## Methods

### Design

An observational study of all candidates who entered the Utrecht selection procedure between April 2008 and April 2010 was conducted.

### Selection procedure

After national registration, the selection for Dutch GP training is conducted locally at the department of each candidate’s choice. The local selection committee decides which candidates are invited to the interview using criteria such as mastery of the Dutch language and the quality of motivation expressed in their letters of application. Each member of the selection committee, which consists of a staff member, a GP trainer and a GP trainee, independently assesses the qualities of the candidates, including their motivation, orientation on the job, learning needs and personal attributes, after a personal interview with a duration of 30 to 45 minutes [1]. All assessors receive written and oral training at the beginning of the selection procedure to learn how to question and score these qualities.

Sample questions from the semi-structured interview

Motivation to become a GP:

Why did you choose to become a GP among all of the specialisations?

Did you consider other specialisations?

What type of GP do you want to become in the future?

What is the relevance of your CV?

Learning needs/learning styles of candidates:

What are your strengths and weaknesses in learning?

What methods are helpful for you in developing your knowledge, skills and attitude?

What is your experience with group sessions, video assessments, OSCEs and other activities in relation to your own learning?

Orientation/insight on the job as a GP:

What do you know about the range of tasks/job responsibilities of a GP?

What do you know about collaboration with other disciplines?

What medical journals did you read to prepare for postgraduate training?

What is your future vision as a GP?

Personal attributes in relation to clinical performance *(Please provide an example)*:

How do you make decisions?

How do you take responsibility?

How do you cope with pressure and uncertainty?

How do you provide and handle feedback?

### Data collection

All data were derived from the Utrecht postgraduate GP training. Ethical approval for routinely gathered data was not mandatory at the time this study was conducted. Therefore, we executed the study according to the ‘code of conduct’ for the use of personal data in scientific research. Before data processing, all data were clerically anonymised.

Individual characteristics were age at the moment of selection (in years); gender (male versus female); region of medical school (north- west Europe versus elsewhere); past clinical performance after graduation (less than one year; more than one year) and the number of times of application (first time versus second or third time). Candidates’ qualities (motivation, orientation on the job, learning needs and personal attributes) were independently rated on a three point scale by the three members of the selection committee (below standard (1), standard (2), above standard (3)).

The outcome measure was: admission into postgraduate GP training.

### Analysis

We first explored differences between the characteristics and qualities of candidates who applied for the first time versus those who applied for the second or third time. Subsequently, we described reliability aspects, with mean quality scores (SD) according to the three groups of assessors. Inter-rater reliability was estimated for each quality with intraclass correlation coefficients (ICC), calculated for all assessors and any combination of two assessors [9]. Associations between the characteristics and qualities and admission into postgraduate training were estimated with log binomial models. Therefore they are reported as relative risks [10]. In case of missing data (1,3% of data values), mean values or modal category scores were imputed [11].

Some candidates (N = 50) were included more than once in our study population due to consecutive selection procedures. We thus controlled whether the association between determinants and the outcome differed for those who applied for the first time (model 1) versus those who applied for the second or third time (model 2). We computed the linear predictor for candidates who applied for the second and the third time based on the analysis of candidates who applied for the first time [12-14]. This linear predictor was subsequently analysed as a single determinant in model 2. If the results from model 1 are valid for second and third time candidates, the regression coefficient of this linear predictor in model 2 will be close to 1. The analysis was done in SPSS version 17 and SAS version 9.2.

## Results

### Candidates’ characteristics

Three hundred ninety four candidates applied for the postgraduate GP training between April 2008 and April 2010 in Utrecht. Twenty-six were rejected based on their letter of application. Candidates who applied more than 3 times were excluded (N = 3). A total of 365 candidates were included in the study population: 264 applied for the first time, 87 for the second time and 14 for the third time. One fourth of the candidates were male, the mean age was 29.7 years (SD 4.9) and 94.5% followed medical school in north west Europe (Table 1). The group who applied for the second or third time was older and had more clinical experience. The mean score of the candidates’ qualities varied from 2.0 (orientation on the job) to 2.3 (motivation). Candidates who applied for the second or third time had approximately the same scores on personal qualities as those who applied for the first time, with one exception: they had lower scores on personal attributes (Table 1).

**Table 1:** Baseline characteristics of candidates

**Individual characteristics**	**1st time application**	**2nd/3rd time application**	**Total**
	**N = 264**	**N = 101**	**N = 365**
Gender male, N (%)	68 (25.8)	30 (29.7)	98 (26.8)
Age, mean in years (SD)	29.2 (4.7)	31.0 (5.2)	29.7 (4.9)
Medical school NW Europe, N (%)	251 (95.1)	94 (93.1)	345 (94.5)
Past clinical performance < 1 year, N (%)	136 (51.5)	34 (33.7)	170 (46.6)
**Candidates’ qualities**			
Motivation, total mean score (SD)	2.3 (0.6)	2.4 (0.5)	2.3 (0.6)
Orientation on the job, total mean score (SD)	2.0 (0.5)	2.1 (0.5)	2.0 (0.5)
Learning needs, total mean score (SD)	2.3 (0.5)	2.2 (0.6)	2.2 (0.5)
Personal attributes, total mean score (SD)*	2.3 (0.6)	2.1 (0.5)	2.2 (0.6)
**Admitted**, N (%)	148 (56.1)	58 (57.4)	206 (56.4)

*SD =* standard deviation.

*difference 0.2 (CI 95%: 0.1 – 0.3).

### Reliability

There were almost no differences in mean scores between the three groups of assessors, or in the standard deviation (Table 2). The reliability of the scores among assessors was good, with the lowest score for learning needs (ICC: 0.78 – 0.84). If the assessments of trainees were deleted, the ICC diminished least (ICC: 0.73 – 0.79). The reduction of the ICC was highest (ICC: 0.68-0.75) in case of deleting the assessments of the group of staff members. There were no differences regarding reliability between candidates who applied for the first time versus the second or third time (not shown in a table). There was a moderate to strong association amongst the four qualities (Pearson’s r: 0.40 – 0.64, not shown in a table), indicating that those who scored rather high on motivation did also on orientation on the job, learning needs and personal attributes and vice versa.

**Table 2:** Mean scores (SD) of the assessed qualities according to interviewer; Interrater reliability (Intraclass correlation, ICC)

**Quality**	**Staff member**	**Trainer**	**Trainee**	**ICC**	**ICC without staff member**	**ICC without trainer**	**ICC without trainee**
**N = 365**	**mean(SD)**	**mean(SD)**	**mean(SD)**				
Motivation	2.3 (0.6)	2.3 (0.7)	2.3 (0.6)	0.84	0.75	0.81	0.79
Orientation on the job	2.0 (0.6)	2.0 (0.6)	2.0 (0.6)	0.84	0.75	0.78	0.79
Learning needs	2.2 (0.6)	2.3 (0.6)	2.2 (0.6)	0.78	0.68	0.68	0.73
Personal attributes	2.2 (0.7)	2.3 (0.6)	2.2 (0.7)	0.83	0.71	0.78	0.79

*SD =* standard deviation.

### Predictors

Each of the four candidates’ qualities was independently associated with *being admitted* into the GP training (Table 3), with personal attributes and motivation being the strongest predictors. Individual characteristics, such as age and gender, did not show an association with *being admitted*. We applied the results of the regression analysis of the first application to the candidates who applied for the second or third time, which resulted in a regression coefficient of 0.93 (95% CI 0.72 – 1.15). Therefore, the results in both groups were similar.

**Table 3:** Univariate and Multivariate Relative Risks (95% CI) of being admitted to the GP training

**N = 365**	**Univariate RR (95% CI)**	**Multivariate RR (95% CI)**
Age (in years)	0.95 (0.92 – 0.97)	0.98 (0.96 – 1.00)
Gender (male = ref)	0.87 (0.70 – 1.09)	1.09 (0.92 – 1.28)
Region (NW Europe = ref)	2.33 (1.09 – 5.01)	1.18 (0.65 – 2.14)
Past performance (< 1 year = ref)	1.08 (0.90 – 1.30)	1.12 (0.97 – 1.29)
Motivation	3.18 (2.67 – 3.78)	1.76 (1.46 – 2.12)
Orientation on the job	2.38 (2.03 – 2.79)	1.34 (1.14 – 1.59)
Learning needs	3.20 (2.72 – 3.77)	1.42 (1.17 – 1.73)
Personal attributes	3.09 (2.64 – 3.62)	1.84 (1.54 – 2.19)

*CI =* confidence interval.

ref *=* reference group.

## Discussion

### Summary of main findings

The mean scores and variations in personal qualities awarded by staff members, GP trainers and trainees were nearly the same. The reliability of interview assessments among the three assessors was satisfactory. Exclusion of the assessments of one group (staff member, trainer or trainee) just slightly reduced reliability. Our results show an independent relation between personal qualities, selection criteria, and admission into the postgraduate training; age and gender did not influence the decision.

### Discussion of results

Reviews have shown varying reliability in medical school admission interviews, as previous studies were not primarily designed to investigate reliability, because the format and structure of the interview widely vary and because of assessor bias [2,4,15]. The current study demonstrates a satisfactory level of reliability of the candidates’ quality assessments, which corresponds with more recent studies [16,17]. This may be an effect of structuring the interview and training the assessors, which are factors known to enhance reliability [3-5,15]. The reliability of the interview assessments in this study can be considered satisfactory as well with a view on the duration of the interviews, because reliability of an assessment procedure partly depends on the duration of the procedure [18].

At this time the selection committee consists of three groups of assessors, who conduct assessments from their specific perspectives. Our results show that two assessors would have been sufficient in terms of reliability. This finding is in accordance with other studies that find satisfactory reliability between 2 assessors [19,20]. In general the staff member has the most experience in assessing candidates. This is reflected by the somewhat higher ICC’s of all pairs of assessments in which the staff member’ assessments were included. Extension of the number of instruments, with Multiple Mini Interview (MMI’s) regarding to collaboration, professionalism and doctor patient encounters, and further structuring the interviews, may improve reliability [21,22].

In accordance with our earlier findings candidates’ qualities, such as motivation, orientation on the job, and personal attributes, were independently associated with being admitted [1]. Individual characteristics, such as age and gender did not correlate with the decision of being admitted. These findings are in line with the formal procedure and study by Lumb et al. [1,16], whereas Shaw et al. found that the gender and race of candidates influenced the interview scoring [23].

### Strengths and limitations of the study

By using data on five consecutive selection procedures, it was possible to analyse the assessments of more than 300 candidates. The extent of the group made it possible to determine whether the results differed for candidates who applied for the first time versus candidates applying for the second or third time [1].

This study also has certain limitations. First, the favourable reliabilities may be partly caused by the limited scale width (a three-point scale). However, the literature indicates that the reliability of ratings at the high or low ends of a rating scale is higher than that for the middle levels. Thus, a three-point scale may be as useful as the commonly used five-point scale [8,24]. Controlling the results by calculating the (nonparametric) Kendall’s coefficient of concordance W for the candidates’ quality assessments yielded similar results (coefficient of concordance W for three of the four qualities between 0.75 and 0.77; learning needs: 0.69; all p < 0.05) [25].

Secondly, the correlation between qualities may suggest a halo effect, but this cannot be studied further with these data. The candidates were assessed by various assessors. Therefore, the design did not allow a generalisability analysis, nor did the design provide the opportunity to investigate assessment bias by calculating sources of variance.

## Conclusion

Interview assessments by two representatives of relevant professional groups – a staff member and a trainer - show satisfactory reliability compared with interviews by three representatives. Given this finding and the promising results from the literature of multiple independent assessments in the selection procedures, we plead for a reduction of the number of assessors in the interviews and an extension of the instruments, eg with MMI’s, for a more reliable and valid competence based procedure [8].

## Competing interests

The authors declare that they have no competing interests.

## Authors’ contributions

MIV: initiated the research project, collected data, was involved in the analyses and interpretation of data, and wrote and revised the manuscript. MMK: supervised the design, data collection, analysis and interpretation of data, and contributed in writing and revising the manuscript. NPAZ: performed the statistical analysis, contributed to the interpretation of the data and co-wrote the manuscript. YvdG and RAMJD have been involved in interpreting data and critically revising the manuscript for important intellectual content. All authors have given final approval of the version to be published.

## Pre-publication history

The pre-publication history for this paper can be accessed here:

http://www.biomedcentral.com/1471-2296/14/43/prepub
